# Supplementary material for: Micromotors of MnO2 for the Recovery of Microplastics
Source: Micromachines (Basel). 2024 Jan 17;15(1):141. doi: 10.3390/mi15010141 (PMC10821339; doi:10.3390/mi15010141)
Supplement: Supplementary file 1 [file micromachines-15-00141-s001.zip › micromachines-2745541-supplementary.pdf]

micromachines

Supplementary Material: Micromotors of MnO_2 for the Recovery of Microplastics

Oscar Cervantes [1,2], Claudia Valtierra-Montiel [3], Laura Sampedro-Plata [1], Norberto Casillas [2], Nieves Menendez [1] and Pilar Herrasti [1,*]

[1] Department of Applied Physical Chemistry, Faculty of Sciences, Autonomous University of Madrid, Francisco Tomás y Valiente 7, 28049 Madrid, Spain; ocervantesa@gmail.com (O.C.); laura.sampedrop@estudiante.uam.es (L.S.-P.); nieves.menendez@uam.es (N.M.)
[2] Department of Chemistry, Center of Exact Sciences and Engineering (CUCEI), University of Guadalajara, Marcelino García Barragán 1421, Col. Olímpica, Guadalajara 44430, Jalisco, Mexico; norberto.casillas@academicos.udg.mx
[3] Master's Program in Nanomaterials Science and Technology, Natural and Exact Sciences Division, University of Guanajuato, Noria Alta S/N, Guanajuato 36050, Guanajuato, Mexico; cd.valtierramontiel@hotmail.com
* Correspondence: pilar.herrasti@uam.es

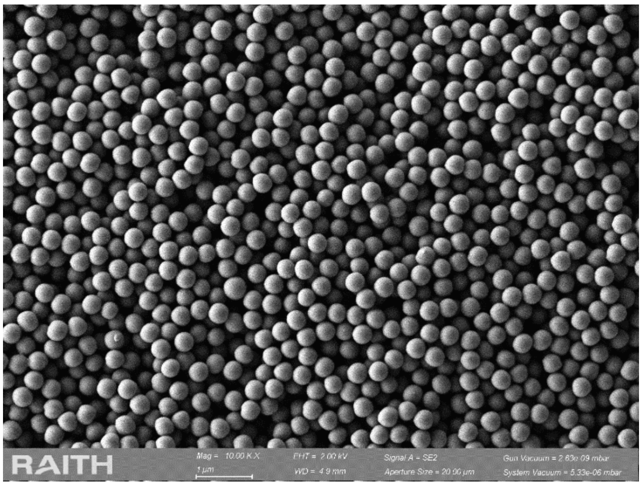

(**a**)

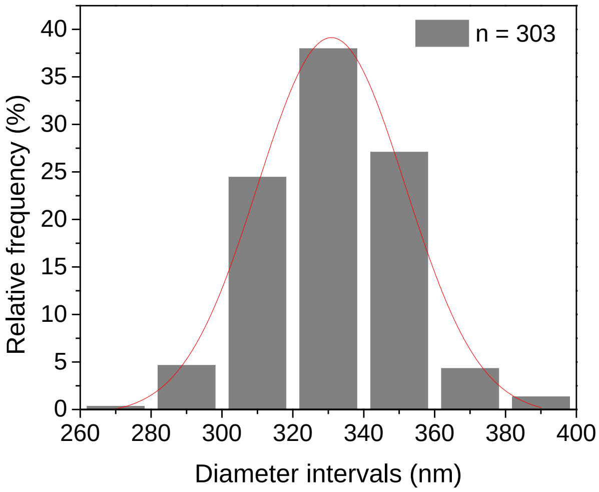

(**b**)

Figure S1. FSEM images of the PS particles (**a**), and their size distribution (**b**).

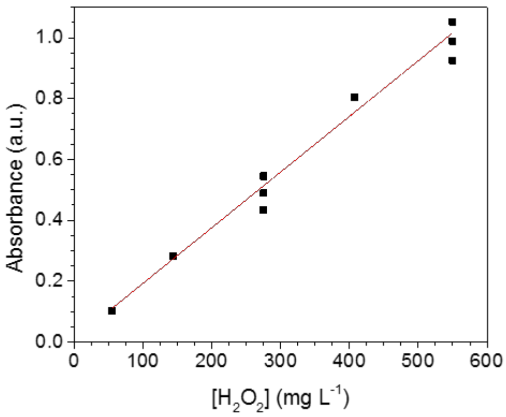

Figure S2. Simple regression and linear fit for Absorbance vs. [H_2O_2].

Table S1. Coefficients and Analysis of Variance for I vs. [H_2O_2].

Parameter	*Least Squares Estimate*	*Standard Error*	*T Statistic*	*P-Value*
**Slope**	0.00185488	0.000033572	55.2508	0.0000

Source	*Sum of Squares*	*Df*	*Mean Square*	*F-Ratio*	*P-Value*
**Model**	5.85499	1	5.85499	3052.65	0.0000
**Residual**	0.0268521	14	0.001918		
**Total**	5.88184	15			

Table S2. Analysis of Variance for %[H_2O_2] remaining in solution vs. %[H_2O_2]$_o$.

Source	*Sum of Squares*	*Df*	*Mean Square*	*F-Ratio*	*P-Value*
**Between groups**	27709.4	2	13854.7	39.43	0.0000
**Within groups**	8432.77	24	351.366		
**Total (Corr.)**	36142.2	26			

Table S3. Analysis of Variance for TOC/TOC_o vs. [PS].

Source	*Sum of Squares*	*Df*	*Mean Square*	*F-Ratio*	*P-Value*
**MAIN EFFECTS**					
**A:PS (mg L^{-1})**	0.002951	2	0.0014755	0.16	0.8520
**B:[H_2O_2]$_o$ (% in wt.)**	0.0903406	2	0.0451703	4.95	0.0194
**INTERACTIONS**					
**AB**	0.0543381	4	0.0135845	1.49	0.2474
**RESIDUAL**	0.16432	18	0.00912891		
**TOTAL (CORRECTED)**	0.31195	26			

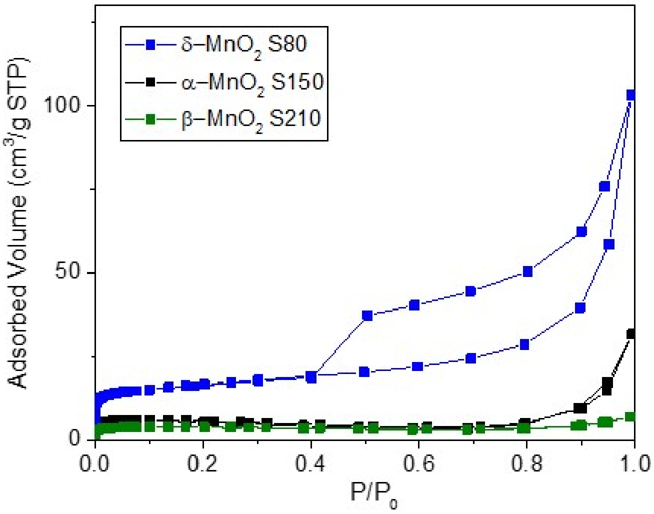

Figure S3. Nitrogen adsorption-desoption isotherms of the three MnO_2 synthesized samples.

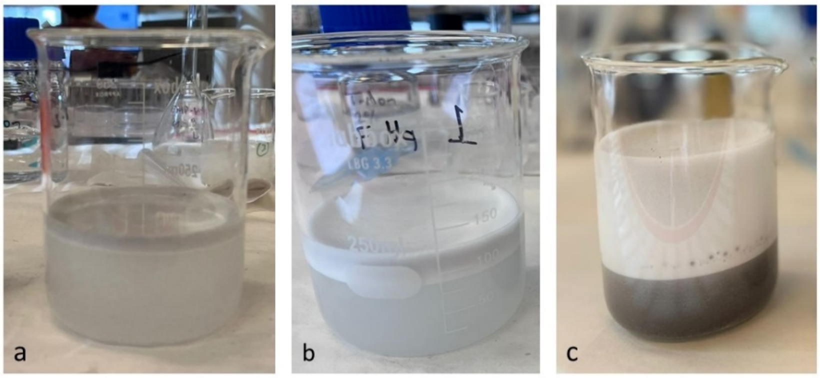

Figure S4. Effect of pH on the amount of foam formed after 5 minutes reaction time, with 0.2 g L^{-1} MnO_2, 20 mg L^{-1} MP, 1.6% H_2O_2 and 0.01% Triton-X. (**a**) pH 3, (**b**) pH 7, and (**c**) pH 9.

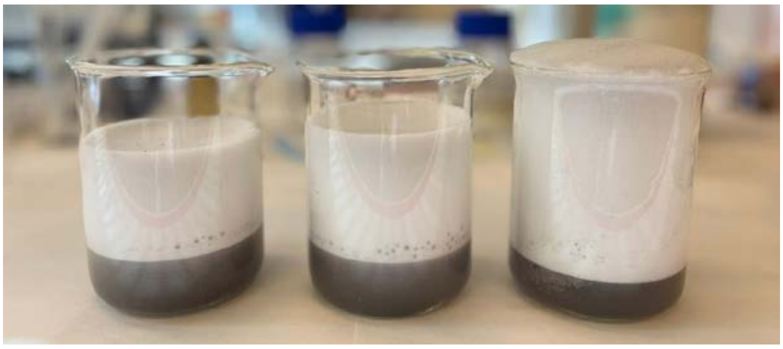

(**a**) (**b**) (**c**)

Figure S5. Effect of H_2O_2 concentration on the foam formation 5 min after the reaction started. 0.2 g L^{-1} MnO_2, 20 mg L^{-1} MP, pH 9 and 0.01% Triton-X. (a) 1.6% H_2O_2, (b) 3% H_2O_2 and (c) 6% H_2O_2.
